# Effects of triclosan on aquatic invertebrates in tropics and the influence of pH on its toxicity on microalgae

**DOI:** 10.1007/s11356-016-7302-0

**Published:** 2016-08-20

**Authors:** Jidapa Khatikarn, Kriengkrai Satapornvanit, Oliver R. Price, Paul J. Van den Brink

**Affiliations:** 1 Department of Aquatic Ecology and Water Quality Management, Wageningen University, P.O. Box 47, 6700 AA Wageningen, The Netherlands; 2 Department of Fishery Biology, Faculty of Fisheries, Kasetsart University, Chatuchak, Bangkok, 10900 Thailand; 3 Safety and Environmental Assurance Centre, Unilever, Colworth Science Park, Sharnbrook, MK44 1LQ Bedfordshire, UK; 4 Alterra, Wageningen University and Research Centre, P.O. Box 47, 6700 AA Wageningen, The Netherlands

**Keywords:** Antimicrobial, Triclosan, Acute toxicity, Aquatic, Invertebrates, pH, Tropics

## Abstract

The antimicrobial triclosan (TCS) has been detected in household wastewaters (untreated and treated) and receiving environments across the globe. The toxic effects of TCS on temperate standard aquatic test organisms have been widely reported with microalgae being the most sensitive. However, environmental differences between tropical and temperate regions may have selected different trait compositions between these two regions, which in turn may lead to a difference in species sensitivity. Therefore, additional information is required to better characterize risks to organisms in tropics and ensure biodiversity in these regions is not adversely impacted. This study aims to supplement existing TCS toxicity data with five aquatic invertebrates found in tropics and to compare the sensitivity between aquatic invertebrate species from tropical and temperate regions. In addition, the effect of pH on the toxicity of neutral and ionized forms of TCS to microalgae (*Chlorella ellipsoidea*) was investigated. The reported 96-h LC50 values for the studied invertebrate species ranged from 72 to 962 μg/L. There was no significant difference between the sensitivity of aquatic invertebrate species from tropical and temperate regions. EC50 values for *C. ellipsoidea*, with and without pH buffer, were significantly different. The findings of this study can be used to support site-specific water quality criteria and environmental risk assessment for TCS in tropical regions. However, further chronic and semi-field experiments with TCS could potentially enable a refined assessment of direct and indirect effects on tropical aquatic communities and further explore functional endpoints of tropical ecosystems.

**Electronic supplementary material:**

The online version of this article (doi:10.1007/s11356-016-7302-0) contains supplementary material, which is available to authorized users.

## Introduction

Triclosan (5-chloro-2-(2,4-dichlorophenoxy)phenol, TCS), an antimicrobial agent used in some personal care products, has been detected in household wastewater discharge and receiving environments including regions with poor connectivity to wastewater treatment facilities (Zhao et al. 2010; Ramaswamy et al. 2011; Zhang et al. 2015). The aquatic toxicity of TCS has previously been reported with acute toxicity values for invertebrates ranging from 73.4 to 2890 μg/L, for *Ampelisca abdita* and *Chironomus plumosus*, respectively (Ishibashi et al. 2004; Dussault et al. 2008; Kim et al. 2009; Perron et al. 2012; Wang et al. 2013). Additionally, previous studies reported that microalgae species are the most sensitive species to TCS with chronic toxicity values ranging from 1.4–3.5 μg/L (Orvos et al. 2002; Delorenzo et al. 2008). The majority of previous studies have explored effects on standard aquatic test organisms typical of temperate regions. However, a difference in environmental filtering (i.e., temperature, % organic matter) in the tropical compared to the temperate region can create a different trait composition between these two regions, which in turn may lead to a difference in species sensitivity which tropical species might be more or less sensitive than temperate species (Rubach et al. 2012; Rico and Van den Brink 2015). Therefore, there is a need to obtain additional toxicity data for non-standard test organisms in tropical regions to explore potential impacts on the biodiversity of these regions. Significant differences between sensitivities among tropical and temperate species have previously been reported for some chemicals although for the majority, no differences were found (Kwok et al. 2007; Daam and Van den Brink 2010; Rico et al. 2011). The differences in species sensitivities to 18 chemicals between tropical and temperate freshwater species using species sensitivity distribution (SSD) comparisons were demonstrated in Kwok et al. (2007). For six out of 18 studied chemicals (ammonia, arsenic, zinc, chlorpyrifos, chlordane, and phenol), the tropical species were likely to be more sensitive than the temperate species. For most metals, the temperate species were likely to be more sensitive than the tropical species. In another study, Amazonian aquatic invertebrates were demonstrated to be significantly less sensitive to carbendazim than temperate invertebrates but Amazonian arthropod sensitivity to malathion was found to be similar to the sensitivity of temperate arthropods (Rico et al. 2011). Although the implications of using temperate toxicity data for ecological risk assessment of chemicals in tropical regions has previously been studied, such an assessment has not been conducted yet for TCS. Many historic toxicity studies have not adequately considered the bioavailability of TCS in test media. TCS is a weak acid with p*K*_a_ of 8.1 (Roberts et al. 2014) and can rapidly undergo photolysis (degradation half-life <30 min under sunlight irradiation, Tixier et al. 2002). The neutral form of TCS, at lower pH, has been reported to be more toxic than its ionized form (Rendal et al. 2011). The effect of test media pH on TCS toxicity for microalgae has previously been reported for *Scenedesmus subspicatus* in which toxicity increases with decreasing pH until all TCS is present in an unionized state, then further decrease in pH has no influence as TCS becomes fully neutral at pH 6 and fully ionized at pH 10 (Roberts et al. 2014). Therefore, there is still a need to investigate these effects for other algal species. This study aims to supplement TCS toxicity database on aquatic invertebrate taxa in tropical regions and to compare the sensitivity between aquatic invertebrate species from tropical and temperate regions. In addition, the effect of pH on TCS toxicity to microalgae was investigated to compare the toxicity between neutral and ionized forms of TCS.

## Materials and methods

### Acute toxicity tests with tropical aquatic invertebrates

The acute toxicity of TCS was on five freshwater invertebrate species found in tropics: Ephemeroptera (*Baetis* sp.), Anostraca (*Branchinella thailandensis*), Oligochaeta (*Tubifex tubifex*), Trichoptera (*Leptocerus* sp.), and Decapoda (*Macrobrachium lanchesteri*). Details of the test organisms, toxicity test endpoints, and test conditions are provided in Table 1. Species belonging to two of the taxa tested (*Baetis* sp. and *Leptocerus* sp.) also occur in temperate regions, but unfortunately, due to a lack of identification keys, we could not evaluate whether we have tested a uniquely tropical species or one that has a cosmopolitan distribution and one species tested (*T. tubifex*) is cosmopolitan. However, these tests differed with a temperate test in two aspects. Firstly, these tests were performed under a tropical condition (the test temperature was around 29 °C), and the higher test temperature may increase the bioavailability of chemicals causing the species tested in tropics to be more sensitive (Kwok et al. 2007). Secondly, the organisms were grown in tropical environment, so although genotypically they are the same as their temperate counterpart, they will be different phenotypically. All experiments were performed in the Fishery Biology laboratory at Faculty of Fisheries, Kasetsart University, Bangkok, Thailand. All species were acclimatized in laboratory conditions for at least 48 h prior to the tests. Triclosan, C_12_H_7_Cl_3_O_2_, 99.8 % Purity (HPLC), was purchased as Irgasan from Sigma-Aldrich. TCS stock solutions were made by dissolving TCS in acetone, and test mediums were prepared by diluting the stock solutions in a filtered water. To spike the stock solutions, the volume of acetone added in the test medium never exceeded 0.1 mL/L. Blank controls and solvent controls were performed in three replicates for each endpoint observation. The studied endpoints were immobility for 24-h test of *B. thailandensis* nauplii and mortality for other invertebrate species. Immobility of the tested nauplii was considered when no movement was observed after three-time repeated tactile stimulation with a laboratory needle. The wait period to confirm the immobility of nauplii was 3 s between each tactile stimulation. The observations of immobility and mortality of the nauplii were done under a microscope. The effects of TCS on the studied endpoints were recorded every 24 h throughout the 96-h exposure periods except for the *B. thailandensis* where the effect on mortality was assessed every 48 h during the medium renewal which was performed every other day (Table 1). The test was considered valid when the immobilization or mortality observed in the controls was 10 and 20 % or less at 24 and 96 h, respectively. These values are based on the acceptance criteria of the OECD protocols, which is 10 % for the 24-h *Daphnia* sp. test (OECD 2004) and 15 % for the 48-h *Chironomus* sp. test (OECD 2011). The tests were conducted in glassware to avoid adsorption of TCS to plastic media (Koc et al. 2014). Moreover, the tests were performed in complete darkness to avoid the photolysis of TCS except for the 96-h *B. thailandensis* where the photoperiod was 12 h of natural light as the absence of light affects the growth and survival of *B. thailandensis*. Test methods were based on OECD standard protocol for toxicity testing with *Daphnia magna* (OECD 2004). However, some environmental conditions were adapted to tropical ecosystems.

**Table 1:** Characteristics of the test organisms, toxicity test endpoints, and test conditions

Species	Organism characteristics		Test conditions										
	Life stage	Origin^a^	Endpoint	Test duration (h)	System^b^	Water volume (mL)	Replicates (*n*)	Number per replicate	Aeration	Feed	Water temp (°C)	DO (mg/L)	pH
*Baetis* sp.	Larva	A	Mortality	96	Static	500	3	10	Yes	No	29.8 ± 0.8	5.7 ± 0.2	7.6 ± 0.4
*Branchinella thailandensis*	Nauplii 24-h age	B	Mortality	96	Static-renewal	600	3	10	Yes	Yes^c^	28.5 ± 1.0	6.8 ± 0.7	6.6 ± 0.3
	Nauplii <12-h age	B	Immobility	24	Static	5	10	3	No	No	27.9	6.9	7.0
	Nauplii <12-h age	B	Mortality	24	Static	5	10	3	No	No	27.9	6.9	7.0
*Tubifex tubifex*	Adult	B	Mortality	96	Static	300	3	10	Yes	No	28.2 ± 0.6	6.6 ± 0.3	7.0 ± 0.2
*Leptocerus* sp.	Larva	A	Mortality	96	Static	500	3	8	Yes	No	29.6 ± 0.6	5.8 ± 0.3	7.6 ± 0.4
*Macrobrachium lanchesteri*	Adult	B	Mortality	96	Static-renewal	2000	3	10	Yes	No	29.6 ± 0.7	7.6 ± 0.8	7.7 ± 0.4

^a^A: collected from unpolluted site of Phetchaburi River, Thailand; B: purchased from fish retailers in Thailand

^b^Static: the test mediums were not renewed during the test period; Static-renewal: the test mediums were renewed every other day.

^c^ *Branchinella thailandensis* were fed with *Chlorella ellipsoidea* 10^6^ cell/mL every other day after renewing the test mediums.

### Effect of pH on TCS toxicity to microalgae

The effect of pH on TCS toxicity on the growth of *Chlorella ellipsoidea* (72 h) was assessed. Laboratory axenic cultures of *C. ellipsoidea* were obtained from the Inland Feed Research Institute, Department of Fisheries, Ministry of Agriculture and Cooperatives, Thailand. The culture was inspected for contamination on a weekly basis, and only log-phase algae were used for the toxicity tests. Two experiments were conducted in accordance with OECD guideline 201 (OECD 2006). One followed the guideline without modifications, but a second was conducted with two modifications adapted from the experiment of Roberts et al. (2014), including (1) pH control using 4.5-mM Tris buffer at target pH level of 7.5 and (2) an acclimatization period of 2 × 72 h in the algal medium contained Tris buffer prior to the test. Tris buffer, or Tris(hydroxymethyl)methylamine, NH_2_C(CH_2_OH)_3_, ≥99.8 % Purity, was purchased from UNIVAR. A preliminary test was also performed to assess the effect of Tris buffer on the growth of algae prior performing the actual tests. The tests were conducted with an application of ultraviolet (UV) covers on the lights to avoid photolysis of TCS. Both tests were performed in 250-mL glass Erlenmeyer flasks containing 100 mL of test medium, modified BG-11 (Anderson 2005), and spiked with TCS stock solution in acetone. The volume of acetone added in the test medium never exceeded 0.1 mL/L. The algal test without Tris buffer was performed with the initial cell density of 10^6^ cell/mL according to Andrieu et al. (2015) who studied the effect of antibiotics on *Chlorella* sp. However, we found that the algae growth in the control cultures was a bit less than the OECD recommendation (<16-fold) within the 72-h test period. Therefore, the initial cell density of the algal test with Tris buffer was adjusted to 5 × 10^4^ cell/mL following the OECD guidelines and Roberts et al. (2014) that resulted in algae growth in the control cultures > 16-fold. Each test consisted of six TCS concentrations (0.5, 1.2, 3.1, 7.8, 19.5, and 48.8 μg/L) in three replicates. Blank, solvent, and buffered controls were performed in six replicates for each endpoint observation. The temperature in the media during the test period was 24 ± 2 °C (average ± max, min). During the acclimatization and 72-h test periods, *C. ellipsoidea* were exposed to a continuous light intensity of 4000 lux. The tested flasks were shaken at approximately 100 rpm to keep the algal cells in suspension and to ensure sufficient gas exchange. The algal cell density was measured after 24, 48, and 72 h by counting cell number under a microscope. The pH of the tested mediums were measured at the beginning and at the end of the tests. An average specific growth rate was calculated in accordance with OECD guideline 201 (OECD 2006). To verify the exposure concentrations, a 3-mL pooled sample of test media was taken from each replicate of the two highest test concentrations at the beginning and at the end of the tests.

### Chemical analyses

To verify the exposure concentrations, 3-mL sample of test media was taken from one replicate of the lowest and highest test concentrations at the beginning and at the end of each test. Two-milliliter subsamples of the test media from the invertebrate tests were transferred to 2-mL amber vials and allowed to settle for an hour in the dark, then 0.5-mL supernatant was taken for TCS determination by high-performance liquid chromatography (HPLC). The samples from algae tests required a use of liquid-liquid extraction method to remove microalgae cells from the samples. The aqueous TCS cannot be directly filtered through any filtered material as TCS can bind or adsorb into it. Therefore, the samples needed to be prepared in an organic solvent before the filtration. Firstly, the samples were extracted three times by dichloromethane (DCM, 3 × 300 μL). Secondly, the extracts were transferred into amber vials and then dried with nitrogen gas. Then, if the sample was relatively clean, it was re-dissolved in a mixed solution of 10 % (*v*/*v*) of methanol in Milli-Q water. If the sample contained a lot of suspension, it was re-dissolved in 1-mL methanol and filtered through 0.22 μm organic syringe filter. The known volume filtrate was subsequently transferred to another 2-mL amber vial and dried with nitrogen gas and finally re-dissolved in a solution of 10 % (*v*/*v*) of methanol in Milli-Q water prior to HPLC analysis.

TCS was analyzed on the HPLC, Agilent 1260 Infinity series equipped with an UV detector. A Zorbax Eclipse Plus-C18 column (150 × 4.6 mm, 5 μm) was used for the separation. The column temperature was set at 30 °C. Acetonitrile and 0.1 % acetic acid solution (70:30, *v*/*v*) were used as the mobile phase. The injection volume was 100 μL and the flow rate was set at 1 mL/min. The UV wavelength for TCS detection was 205 nm. The limit of quantitation for TCS was 5 μg/L. The recovery of TCS analyses ranged from ca. 85–95 %.

### Statistical analysis

The effect and lethal concentrations for the 10 and 50 % of the test organisms (EC10/50 or LC10/50) were derived after a number of different exposure periods as described in Table 1 for invertebrates and after 72 h for microalgae. The 95 % confidence interval (CI) was calculated by means of log-logistic regression using GenStat 17th edition (VSN International Ltd., Oxford, UK) as describe in Rubach et al. (2011).

### Comparison of TCS sensitivity between tropical and temperate invertebrate species

Acute toxicity data for aquatic invertebrates were collected from original publications and toxicity database (http://cfpub.epa.gov/ecotox/). The data selection followed those outlined by Maltby et al. (2009). The selected endpoints, with the test duration between 1 and 7 days, were median lethal concentration (LC50) or median effect concentration (EC50) for effects on immobility. Moreover, the distinction between the tropical and temperate organisms was done according to the location where the test organisms were collected or grown. Organisms collected between 23.5° N and 23.5° S of the equator were classified as tropical, while those collected outside this region were classified as temperate (Kwok et al., 2007). The sensitivity difference of tropical and temperate aquatic invertebrates was compared using the species sensitivity distribution concept (SSD) (Rico et al. 2010). The SSD curves were fitted using the ETX 2.0 software (Van Vlaardingen et al. 2004). Anderson–Darling goodness of fit test was performed for log-normality test, with normality of the toxicity data assumed at *p* ≥ 0.05 (Posthuma et al. 2002). Significant differences between tropical and temperate SSDs were assessed by two-sample Kolmogorov–Smirnov test using SPSS 22.0 statistical package. The hazardous concentrations for 5 and 50 % of the invertebrate species (HC5 and HC50, respectively) were calculated (Aldenberg and Jaworska 2000).

## Results and discussion

The measured test concentrations of the tested media were maintained within ±20 % of nominal or measured initial concentrations throughout the tests, so the calculations of ECs and LCs results were based on the nominal concentrations. The measured medium concentrations and percent dissipations are shown in the supplementary information.

### Toxicity test results of five tropical aquatic invertebrates

The average mortality and immobility in blank and solvent controls did not exceed 10 and 20 % in the 24- and 96-h tests, respectively (Table 2). The results of the toxicity tests, L(E)C10 and L(E)C50 values (in μg/L) and their 95 % confidence intervals (CI), are shown in Table 2. Results of the acute toxicity tests showed that *Baetis* sp. with a 96-h LC50 of 72 μg/L was the most sensitive species to TCS followed by *B. thailandensis* (100 μg/L), *T. tubifex* (259 μg/L), *Leptocerus* sp. (760 μg/L), and *M. lanchesteri* (962 μg/L). In addition, this study demonstrated that a 72-h exposure time was sufficient to test these invertebrates. The 72-h LC50 values of *Baetis* sp., *T. tubifex*, *Leptocerus* sp., and *M. lanchesteri* were 96, 266, 866, and 1005 μg/L, respectively, which was not significantly different from the 96-h LC50 values. *B. thailandensis* test was only evaluated at 48-h intervals, and its 48-h LC50 (130 μg/L) was not significantly different from the 96-h LC50. The LC50 values at different exposure times for each species are given in the supplementary information. The result of *Baetis* sp. shows that TCS is relatively toxic to Ephemeroptera*.* For TCS, no previous toxicity tests with this taxon have been conducted. However, based on acute toxicity data for other chemicals like insecticides, Ephemeroptera seem to be among the most sensitive aquatic invertebrates (Beketov 2004; Roessink et al. 2013; Van den Brink et al. 2016). Previous studies on *Cloeon dipterum*, a mayfly nymph, demonstrated high sensitivity to the insecticide imidacloprid (Roessink et al. 2013; Van den Brink et al. 2016) and pyrethroids (Rubach et al. 2010). From the vulnerability analysis by Rico and Van den Brink (2015), some Ephemeroptera taxa were found to be vulnerable to all insecticides, and a good correlation was found between mode of respiration (having gills) and sensitivity. Having gills was also positively correlated with the uptake of and herewith the sensitivity of aquatic invertebrates to the insecticide chlorpyrifos (Rubach et al. 2012). However, to our knowledge, no chemical with a similar mode of action to TCS has previously been tested on Ephemeroptera. Moreover, due to the hydrophobic nature of TCS (log *K*_ow_ > 4) (Dhillon et al. 2015), the dominating mechanisms of chemical uptake by aquatic species are likely to be through open body surfaces, essentially gills, and via the diet. This is a possible explanation for the relatively high sensitivity of the fairy shrimp (*B. thailandensis*) to TCS, with a 96-h LC50 of 100 μg/L, as the test was performed with algal feeding. However, for the fairy shrimp nauplii, the 24-h EC50 and 24-h LC50 were 402 and 522 μg/L, respectively. These results are in accordance with a previous study of Kim et al. (2009) where a 24-h EC50 (immobility) of TCS on *Thamnocephalus platyurus* of 470 μg/L was reported. Conversely, the result of Oligochaeta species in the present study is not consistent with previous studies. In this study, *T. tubifex* is more sensitive to TCS (96-h LC50 of 259 μg/L) than reported by Wang et al. (2013) for *Limnodrilus hoffmeisteri* (96-h LC50 of 2046 μg/L). This could be because the body size of *T. tubifex* is much smaller than *L. hoffmeisteri*, but probably is the effect of a larger fraction of neutral TCS present in the *T. tubifex* test as it was performed at pH 7.0 ± 0.2 while the *L. hoffmeisteri* test was performed at pH 8.0 ± 0.2 (Roberts et al. 2014). For the test with Trichoptera, *Leptocerus* sp. showed low sensitivity to TCS, as the tested larvae were still in their cocoons during the chemical stress event, possibly affecting the TCS exposure and effects (Rico et al. 2015). *M. lanchesteri*, adult freshwater shrimp, was the least sensitive species to TCS with a 96-h LC50 of 962 μg/L, and this sensitivity was in accordance with a previous study on another adult freshwater shrimp species that reported a 96-h LC50 for *Neocaridina denticulata sinensis* of 772 μg/L (Wang et al. 2013). However, saltwater Crustacea species seem to be more sensitive to TCS than the freshwater species. Previous studies reported that 96-h LC50 for *Palaemonetes pugio*, *Americamysis bahia*, and *Ampelisca abdita* were 305, 74.3, 73.4 μg/L, respectively (Delorenzo et al. 2008; Perron et al. 2012).

**Table 2:** Lethal and effective concentration values of triclosan for invertebrates and microalgae

Species	Endpoint	Test duration (h)	Nominal concentration (μg/L)	% Mortality in the control	% Mortality in the solvent control	L(E)C10 in μg/L (CI 95 %)	L(E)C50 in μg/L (CI 95 %)	Slope (L/μg)
Microalgae								
*Chlorella ellipsoidea*								
with pH buffer	Biomass	72	0.5, 1.2, 3.1, 7.8, 19.5, 48.8	NC	NC	2.6 (2.3–3.0)	4.3 (3.8–4.8)	−4.38
without pH buffer	Biomass	72	0.5, 1.2, 3.1, 7.8, 19.5, 48.8	NC	NC	14.7 (11.8–18.3)	28.9 (25.4–32.8)	−3.25
Invertebrates								
*Baetis* sp.	Mortality	96	31.2, 62.5, 125, 250, 500	20.0	13.3	17 (7–41)	72 (48–107)	1.54
*Branchinella thailandensis*	Mortality	96	15.6, 31.2, 62.5, 125, 250	16.7	6.7	46 (27–78)	100 (78–128)	2.79
	Immobility	24	31.2, 62.5, 125, 250, 500, 1000	6.7	13.3	186 (104–334)	402 (307–527)	2.85
	Mortality	24	31.2, 62.5, 125, 250, 500, 1000	0	10.0	472 (449–496)	522 (499–547)	21.53
*Tubifex tubifex*	Mortality	96	62.5, 125, 250, 500, 1000	3.3	3.3	241 (NC)	259 (NC)	30.51
*Leptocerus* sp.	Mortality	96	62.5, 125, 250, 500, 1000	0	0	102 (45–230)	760 (444–1298)	1.09
*Macrobrachium lanchesteri*	Mortality	96	200, 400, 800, 1500, 3000	13.3	3.3	667 (534–832)	962 (838–1105)	5.99

NC: not calculated

### Effect of pH on TCS toxicity to *C. ellipsoidea*

Preliminary tests showed no effect of the Tris buffer on the growth of algae prior to performing the definitive study. The tests with and without Tris buffer had the same average specific growth rates of 0.057 days^−1^. For the test without Tris buffer, the pH of all test and control vessels ranged from 7.2 to 7.3 at the beginning and 8.1 to 10.7 at the end of the test. For the test with Tris buffer, the pH of all test and control vessels was 7.5 at the beginning and ranged from 7.6 to 7.9 at the end of the test. Both tests showed that the algae cell densities reduced significantly with increasing concentrations of TCS. The 72-h EC50 of the algal test without Tris buffer, 28.9 μg/L, was found to be markedly higher than the 72-h EC50 of the algal test with Tris buffer, 4.3 μg/L. Similar responses in growth were observed during a 48-h exposure time, 48-h EC50 of 28.5 μg/L for without Tris buffer, and 3.5 μg/L for with Tris buffer. For the test with Tris buffer, algae exposed to TCS at 19.5 and 48.8 μg/L for 72 h showed almost no growth as compared to algae exposed to lower concentrations. The significant higher sensitivity of *C. ellipsoidea* tested with Tris buffer could be explained by the occurrence of a higher proportion of neutral TCS in the test, which is more toxic than the ionized form (Rendal et al. 2011). The effects of pH on TCS toxicity to *Scenedesmus subspicatus*, a freshwater microalgae, was previously studied by Roberts et al. (2014). They reported that TCS toxicity increased with decreasing pH, with EC50 values of 3.5, 9.1, and 41 μg/L at pH 7.0, 8.0, and 8.5, respectively. With pH control, the sensitivity of *C. ellipsoidea*, 72-h EC50 of 4.3 μg/L at pH 7.5, was in accordance with the EC50 values of *S. subspicatus*. The pH-dependent speciation of TCS calculated based on Handerson-Hasselbalch equation is shown in the supplementary information (Po and Senozan 2001). In contrast, a study by Wu et al. (2009) showed a lower sensitivity of saltwater *Chlorella* sp. to TCS with a 96-h EC50 of 65 μg/L. However, their study was performed without UV covers and without pH control, which is likely to have resulted in loss of TCS via photolysis and increasing pH during the study leading to a higher proportion of ionized TCS. Moreover, the reason for the differences in sensitivities between different microalgal species still remains unclear but may be related with the fact that TCS may react with several target sites in different microalgal species (Franz et al. 2008). These results confirm the need for similar pH control and UV cover methods to be implemented for use in algal toxicity tests for ionizing and photolytic chemicals; there is a need that these studies are conducted under realistic environmental conditions (i.e., pH of receiving environments, continuous chemical concentrations in water bodies). Alternatively, the toxicity of ionizing chemicals could be expressed in their toxic speciation form, e.g. unionised TCS. The use of UV cover is also necessary to avoid photolysis in test media. This enables laboratory concentrations to be maintained and better reflect in river concentrations of chemicals discharged with household wastewater.

### Comparison of TCS sensitivity between tropical and temperate aquatic invertebrate species

**Fig. 1:**
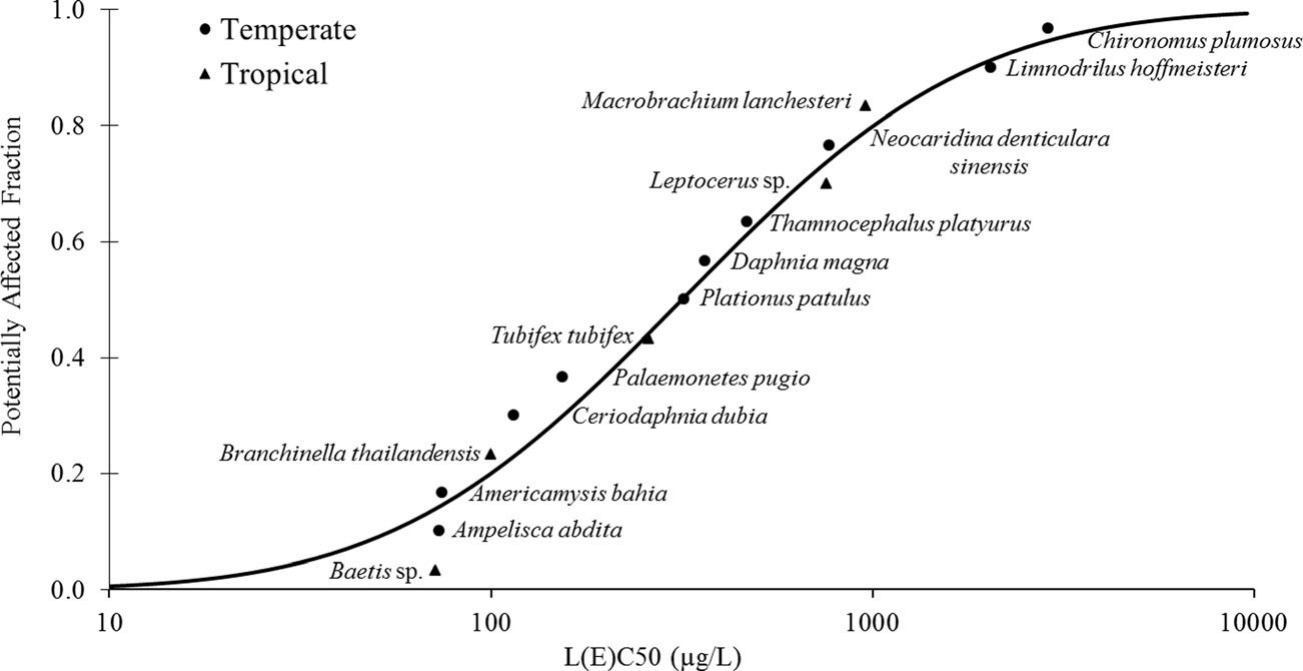
Species sensitivity distribution of tropical and temperate aquatic invertebrate species for triclosan

The selected data (Table 3) for the generation of SSD curves of tropical and temperate aquatic invertebrate species (or cosmopolitan species tested or grown under tropical or temperate conditions) were performed following the study of Maltby et al. (2009) and classified following the study of Kwok et al. (2007). Though there were 10 temperate aquatic invertebrates selected for the generation of the SSD curve, there was no acute toxicity data of tropical aquatic invertebrates found in the original publications and the toxicity database. Therefore, the tropical data used in the comparison originated only from this study (*N* = 5). Both data sets passed the Anderson-Darling goodness of fit test (*α* = 0.05) indicating that the studied toxicity datasets satisfied the normally distributed conditions. The results of two-sample Kolmogorov-Smirnov tests did not reveal significant difference between the sensitivity distributions of tropical and temperate aquatic invertebrate species (*n*_1_ = 10, *n*_2_ = 5, *p* = 0.999). The SSD curve of tropical and temperate aquatic invertebrate species is shown in Fig. 1. The median HC5 and HC50 values for tropical and temperate SSDs with their lower (95 %) and upper (5 %) confidence limits are shown in Table 4. For TCS, median HC5 and HC50 values for tropical aquatic invertebrates were found to be similar to the values calculated from the temperate species. Therefore, the HC5 of 41.1 μg/L was calculated from all available acute toxicity data for aquatic invertebrate species as it is the real representative to derive a PNEC for TCS. Similarly, Wang et al. (2013) also reported that there was no statistically significant difference between the sensitivities of native and non-native species in China to TCS by comparing the SSDs. The results of outdoor microcosm experiments performed by Daam et al. (2009) showed insignificant differences in sensitivities between tropical invertebrate communities in Thailand and temperate communities to carbendazim, a widely used fungicide. Rico et al. (2010) also found that Amazonian and temperate fish and freshwater arthropods show a similar sensitivity to the insecticide parathion-methyl. In addition, Maltby et al. (2005) demonstrated that habitat type and geographical distribution of species do not influence the hazard assessment of chemicals. However, taxonomic composition of species is significant for constructing SSDs for use in risk assessment.

As TCS concentrations have been detected in the tropical and temperate environments, the risk of TCS to the aquatic organisms should be evaluated. The risk estimation of TCS to the aquatic environment studied by Lyndall et al. (2010) was performed using measured and modeled TCS concentrations compared with the chronic SSDs. The results showed that the potential risk of TCS is relatively low in most situations. However, the study of Daam and Van den Brink (2010) indicated that climate related parameters, ecosystem sensitivity, and agricultural practices (in this case, wastewater management practices) differ significantly in tropical regions altering potential risk profiles. For example, direct discharges of untreated household wastewaters in tropical regions can potentially lead to higher input of TCS and other chemicals into waterbodies which could pose more risk to the tropical ecosystems. However, the higher nutrient and higher irradiance levels in the tropics also lead to increased algal biomass contributing to higher pH values compared to temperate regions. The higher irradiance and higher pH are likely to result in greater degradation of TCS via photolysis and increase the prevalence of the less toxic ionized TCS which will potentially reduce the aquatic risk.

**Table 3:** Summary data for species sensitivity distributions (SSDs) of tropical and temperate species for triclosan

Species		L(E)C50, μg/L	Test duration (h)	pH	Reference
Tropical					
*Baetis* sp.^a^	Ephemeroptera	72	96	7.5 ± 0.2	This study
*Branchinella thailandensis*	Anostraca	100	96	7.0 ± 0.2	This study
*Tubifex tubifex* ^a^	Oligochaeta	259	96	6.9 ± 0.2	This study
*Leptocerus* sp.^a^	Trichoptera	760	96	7.2 ± 0.2	This study
*Macrobrachium lanchesteri*	Decapoda	962	96	7.7 ± 1.1	This study
Temperate					
*Ampelisca abdita*	Amphipoda	73.4	96	NR	Perron et al. (2012)
*Americamysis bahia*	Mysida	74.3	96	NR	Perron et al. (2012)
*Ceriodaphnia dubia* ^a^	Cladocera	115	48	8.0 and 8.5	Orvos et al. (2002)
*Palaemonetes pugio*	Decapoda	154	96	NR	DeLorenzo et al. (2008)
*Plationus patulus* ^a^	Ploima	320	48	7.5 ± 0.02	Martinez Gomez et al. (2015)
*Daphnia magna* ^a^	Cladocera	363	48	8.1 and 8.0 ± 0.2	Orvos et al. (2002) and Wang et al. (2013)
*Thamnocephalus platyurus*	Anostraca	470	24	NR	Kim et al. (2009)
*Neocaridina denticulara sinensis*	Decapoda	772	96	8.0 ± 0.2	Wang et al. (2013)
*Limnodrilus hoffmeisteri* ^a^	Oligochaeta	2046	96	8.0 ± 0.2	Wang et al. (2013)
*Chironomus plumosus*	Diptera	2890	96	8.0 ± 0.2	Wang et al. (2013)

NR: not reported

^a^These species also present in both tropics and temperate. The distinguishment was done according to the performed test temperature and the organism collected origin.

**Table 4:** Median hazardous concentrations for 5 and 50 % of species (HC5 and HC50, respectively; μg/L) and their lower (95 %) and upper (5 %) confidence limits, derived from the SSD shown in Fig. 1 for tropical, temperate, and all invertebrate species in μg/L

	Number of species	HC5	HC50
Tropical	5	33.7 (2.0–103.2)	267.2 (88.1–810.8)
Temperate	10	38.2 (8.0–92.6)	345.3 (163.2–730.6)
All species	15	41.1 (14.0–81.8)	317.0 (182.4–551.0)

## Conclusion

This study supplements existing surface water risk assessments for TCS and provides new insights into its toxicity to a range of aquatic invertebrate species in the tropics. Of the species tested, we demonstrate that Ephemeroptera, *Baetis* sp., was the most sensitive and Decapoda, *M. lanchesteri*, was the least sensitive. This study provides useful information for environmental risk assessments of TCS in regions that have tropical aquatic environments. We demonstrate that there was no significant difference between the sensitivity distributions for acute toxicity to TCS for both tropical and temperate aquatic invertebrate species. This implies that the aquatic toxicity data used to support environmental risk assessment of TCS in temperate regions can potentially be extrapolated to support risk assessments in tropical regions. Due to its potential constant discharge via household wastewaters in tropical regions, additional chronic and semi-field experiments with TCS could help further evaluate direct and indirect effects of TCS on tropical aquatic communities and explore impacts on functional endpoints of tropical ecosystems. Such data would help evaluate whether temperate water quality criteria can routinely be used to protect all aquatic ecosystems across the globe. Finally, we confirm the importance of the test design concerning media pH used to assess the toxicity of TCS and highlight that existing risk assessments that rely on historic microalgal toxicity data, which fail to account for differences in pH and/or photolysis during toxicity tests, should be interpreted with caution.

## Electronic supplementary material

ESM 1 (DOCX 22.8 kb)
