# Supplementary material for: Effects of triclosan on aquatic invertebrates in tropics and the influence of pH on its toxicity on microalgae
Source: Environ Sci Pollut Res Int. 2016 Aug 20;25(14):13244–53. doi: 10.1007/s11356-016-7302-0 (PMC5978822; doi:10.1007/s11356-016-7302-0)
Supplement: Supplementary file 1 — (DOCX 22.8 kb) [file 11356_2016_7302_MOESM1_ESM.docx]

**Supplementary information**

**Effects of triclosan on aquatic invertebrates in tropics and the influence of pH on its toxicity on microalgae**

Jidapa Khatikarn^1,2^, Kriengkrai Satapornvanit^2^, Oliver R. Price^3^, Paul J. Van den Brink^1,4^

^1^ Department of Aquatic Ecology and Water Quality Management, Wageningen University, P.O. Box 47, 6700 AA Wageningen, The Netherlands

^2^ Department of Fishery Biology, Faculty of Fisheries, Kasetsart University, Chatuchak, Bangkok 10900, Thailand

^3^ Safety and Environmental Assurance Centre, Unilever, Colworth Science Park, Sharnbrook, MK44 1LQ Bedfordshire, UK

^4^ Alterra, Wageningen University and Research Centre, P.O. Box 47, 6700 AA Wageningen, The Netherlands

**Corresponding author:** Jidapa Khatikarn

e-mail: jidapa.khatikarn@wur.nl

Table S1 The lowest and highest medium concentrations measured at the begining and at the end of the tests

| Species | Nominal concentration (µg/L) | Measured concentration (µg/L) | | % Difference between nominal and measured concentration | % Dissipation |
| --- | --- | --- | --- | --- | --- |
|  |  | Beginning | End |  |  |
| *Chlorella ellipsoidea*; with pH buffer^a^ | 19.5 | 19.2 | 21.5 | 2 | 12 |
|  | 48.8 | 41.1 | 36.4 | 16 | 11 |
| *Chlorella ellipsoidea*; without pH buffer^a^ | 19.5 | 16.6 | 17.1 | 15 | 3 |
|  | 48.8 | 40.3 | 39.2 | 17 | 3 |
| *Baetis* sp. | 31.2 | 37.1 | 36.5 | 19 | 2 |
|  | 500 | 500.0 | 450.9 | 0 | 10 |
| *Branchinella thailandensis* | 15.6 | 16.9 | 15.4 | 8 | 9 |
|  | 250 | 242.2 | 237.2 | 3 | 2 |
| *Tubifex tubifex* | 62.5 | 53.6 | 51.4 | 14 | 4 |
|  | 1000 | 1077.3 | 988.1 | 8 | 8 |
| *Leptocerus* sp. | 62.5 | 67.0 | 66.3 | 7 | 1 |
|  | 1000 | 862.1 | 844.7 | 14 | 2 |
| *Macrobrachium lanchesteri* | 200 | 240.4 | 204.2 | 20 | 15 |
|  | 3000 | 3442.7 | 2824.5 | 15 | 18 |

^a^ Only the two highest medium concentrations were measured due to the limit of quantitation for TCS.

Table S2 The LC50 values in µg/L at different exposure times of each species

| Species | 24h | 48h | 72h | 96h |
| --- | --- | --- | --- | --- |
| *Baetis* sp. | 496 | 252 | 96 | 72 |
| *Branchinella thailandensis* | NM | 130 | NM | 100 |
| *Tubifex tubifex* | 491 | 365 | 266 | 259 |
| *Leptocerus* sp. | 1777 | 2544 | 866 | 760 |
| *Macrobrachium lanchesteri* | 1096 | 1041 | 1005 | 962 |

NM: not measured

Table S3 The pH-dependent speciation of triclosan calculated based on Handerson-Hasselbalch equation

| pH | % Ionised form | % Neutral form |
| --- | --- | --- |
| 8.5 | 72 | 28 |
| 8 | 44 | 56 |
| 7.5 | 20 | 80 |
| 7 | 7 | 93 |
| 6.5 | 2 | 98 |
